# Decursin negatively regulates LPS-induced upregulation of the TLR4 and JNK signaling stimulated by the expression of PRP4 *in vitro*

**DOI:** 10.1080/19768354.2020.1726811

**Published:** 2020-02-17

**Authors:** Muhammad Bilal Ahmed, Salman Ul Islam, Young Sup Lee

**Affiliations:** School of Life Sciences, College of Natural Sciences, Kyungpook National University, Daegu, Korea

**Keywords:** LPS, PRP4, inflammation, decursin, TLR4, JNK

## Abstract

The current investigation was carried out to analyze the correlation of bacterial lipopolysaccharide (LPS) and pre-mRNA processing factor 4B (PRP4) in inducing inflammatory response and cell actin cytoskeleton rearrangement in macrophages (Raw 264.7) and colorectal (HCT116) as well as skin cancer (B16-F10) cells. Cell lines were stimulated with LPS, and the expression of PRP4 as well as pro-inflammatory cytokines and proteins like IL-6, IL-1β, TLR4, and NF-κB were assayed. The results demonstrated that LPS markedly increased the expression of PRP4, IL-6, IL-1β, TLR4, and NF-κB in the cells. LPS and PRP4 concomitantly altered the morphology of cells from an aggregated, flattened shape to a round shape. Decursin, a pyranocoumarin from *Angelica gigas,* inhibited the LPS and PRP4-induced inflammatory response, and reversed the induction of morphological changes. Finally, we established a possible link of LPS with TLR4 and JNK signaling, through which it activated PRP4. Our study provides molecular insights for LPS and PRP4-related pathogenesis and a basis for developing new strategies against metastasis in colorectal cancer and skin melanoma. Our study emphasizes that decursin may be an effective treatment strategy for various cancers in which LPS and PRP4 perform a critical role in inducing inflammatory response and morphological changes leading to cell survival and protection against anti-cancer drugs.

## Introduction

Lipopolysaccharide (LPS) is the major constituent of the outer membrane of Gram-negative bacteria which potently induces inflammatory response through producing various cytokines, inflammatory mediators, and growth factors (Harmey et al. 2002; He et al. 2007; Gassmann et al. 2009; Ikebe et al. 2009). It has been shown that LPS may lead to the induction of systemic inflammation and increases hepatic recruitment of cancer cells *in vivo* (He et al. 2009; Ikebe et al. 2009; Wang et al. 2010). Moreover, it has been shown that LPS-induced inflammation increased the growth of experimental metastases in a murine tumor model, and led to increased angiogenesis *in vitro* and *in vivo* (Wang et al. 2010). In addition to these changes, increased expression of vascular endothelial growth factor, higher vascular permeability and tumor cell invasion/migration were also noted (He et al. 2007; Killeen et al. 2009; Yan et al. 2013). Multiple investigations have revealed that activated Toll-like receptor 4 (TLR4) and the nuclear factor-κB (NF-κB) signaling pathways are involved in elevations of LPS-induced metastasis in each process, including tumor cell adhesion and invasion (Brown and Ruoslahti 2004; Liu et al. 2010). A study reported that LPS upregulated the levels of metadherin, which in turn induced lung metastasis of 4T1 mammary tumor cells (Zhao et al. 2011; Sethi et al. 2012). It is thus postulated that LPS may promote angiogenesis and metastasis; however, the underlying mechanisms remain elusive.

*Angelica gigas*, an important medicinal plant of Umbelliferae family, has been reported to possess various compounds such as coumarins, polyacetylenes, and essential oils (Chi and Kim 1988; Choi et al. 2000; Lee et al. 2002). Among the coumarins, pyranocoumarins such as decursin and decursinol angelate have got considerable attention due to their potent pharmacological characteristics (Ahn et al. 1997; Shehzad et al. 2018). Decursin and its isomer have been shown to exhibit anti-cancer, anti-inflammatory, antiangiogenic, and anti-amnesic activities (Yim et al. 2005; Choi et al. 2012). It has been reported that decursin and DA inhibited pro-inflammatory molecules, such as cytokines, chemokines, and enzymes such as cyclooxygenase-2 and matrix metalloproteinases.

Pre-mRNA processing factor 4B (PRP4), a transcription factor involved in pre-mRNA splicing, was first identified in *Schizosaccharomyces pombe* (Kuhn and Käufer 2003). Previously, it has been reported that PRP4 is involved in reversing anticancer drug-induced cell death in human cancer cell lines through actin cytoskeleton rearrangement and epithelial–mesenchymal transition (EMT) (Islam et al. 2017; Islam, Ahmed, et al. 2018). Herein, we report that LPS induced the activation of PRP4 which resulted in the activation of various cytokines and inflammatory proteins. LPS and PRP4 concomitantly altered cell morphology, which was related to the rearrangement of the actin cytoskeleton. Decursin blocked the LPS and PRP4-induced inflammatory response, and reversed the induction of cell morphological changes. We also struggled to elucidate the underlying mechanism for LPS activating the PRP4.

## Material and methods

### Chemicals and reagents

LPS (cat# L 2630) and decursin were obtained from Sigma-Aldrich (St. Louis, MO, USA). Dulbecco’s modified Eagle’s medium (DMEM), fetal bovine serum (FBS), and penicillin/streptomycin were obtained from Gibco (Carlsbad, CA, USA). PRP4 cDNA open reading frame (ORF) clone HG10835-ACG was purchased from Sino Biological (Wayne, PA, USA), and a PRP8 clone was obtained from Origene (Rockville, MD, USA). Antibodies against PRP4, PRP8, TLR4, NF-κB, I-κBα, E-cadherin, Vimentin, AKT, JNK, ERK, and β-actin were obtained from Santa Cruz Biotechnology (Santa Cruz, CA, USA). A Bradford protein assay kit and electrophoresis reagents were purchased from Bio-Rad Laboratories (Irvine, CA, USA). ECL Prime detection reagent and nitrocellulose membrane were purchased from Amersham (Little Chalfont, Buckinghamshire, UK). Vectashield mounting medium with DAPI (4′,6-diamidino-2-phenylindole) from Vector Laboratories Inc. (Burlingame, CA, USA) was used for staining nuclei. PRP4 siRNA was obtained from Santa Cruz Biotechnology (SC-76257). Lipofectamine® LTX with Plus™ Reagent (Cat# 15338100) and SuperScript III Reverse Transcriptase (Cat# 18080093) were obtained from Invitrogen (Carlsbad, CA, USA). Xfect transfection reagent was purchased from Takara Bio USA, Inc. (Mountain View, CA, USA). JNK inhibitor SP600125 (Cat# tlrl-sp60), and TLR4 signaling inhibitor CLI-095 (Cat# tlrl-cli95) were obtained from InvivoGen (California 92121 USA). All chemicals and products were used as prescribed by the manufacturers.

### Cells culture and treatment

RAW 264.7 cells (ATCC #TIB-71), HCT 116 (ATCC #CCL-24), and B16-F10 (ATCC #CRL-6475) were cultured in Dulbeccòs Modified Eagle Medium (DMEM, Gibco #11995-065), respectively. Both media were supplemented with 10% Fetal Bovine Serum (Gibco #16000-044) and 1% penicillin–streptomycin (Gibco #15140-122). Cell cultures were maintained in a humidified incubator containing 5% CO2 at 37°C. Decursin was dissolved in dimethyl sulfoxide and cells were treated with 10 μM curcumin for 24 h (Islam, Lee, et al. 2018).

### F-actin staining

Alexa Fluor 488 phalloidin was used for F-actin visualization. Briefly, after removal of full growth medium, cells were washed twice with 1X phosphate-buffered saline (PBS) and fixed with 4% paraformaldehyde for 15 min at room temperature. Then, cells were permeabilized with 0.2% Triton X-100 for 5 min, and washed 2–3 times with 1X PBS. Alexa Fluor 488 phalloidin stock solution (6.6 μM in methanol) was diluted to 1:40 with 1% bovine serum albumin (BSA), and added to the cells for 50 min at room temperature in the dark. Cells were washed 5–6 times with 1X PBS and actin cytoskeleton was observed using a ZEISS LSM 800 confocal microscope.

### Western blotting

Total cell lysates were prepared using cell lysis buffer, and the protein concentration was determined using the Bio-Rad Protein Assay. Samples (20–40 μg) were prepared in sodium dodecyl sulfate (SDS) sample buffer, separated via 10% SDS-polyacrylamide gel electrophoresis, and transferred onto a nitrocellulose membrane. The membranes were blocked with 2% albumin (Gendept, USA) solution for 2 h at 4°C. Chemiluminescent signals were developed with Clarity™ ECL Western Blotting Substrate (Bio-Rad) according to the manufacturer’s instructions (Islam et al. 2015).

### Reverse-transcription polymerase chain reaction (RT-PCR)

Total RNA (5 µg) was reverse-transcribed using the SuperScript III First-strand synthesis kit, as has been described previously (Islam et al. 2015; Islam, Ahmed et al. 2018). The synthesized cDNA was incubated with RNase H at 37°C for 1 h. PCR was performed using 2 μL of cDNA and the following primers: following primers: IL-6 Forward 5′-GGTACATCCTCGACGGCATCT-3′ and Reverse 5′-GTGCCTCTTTGCTGCTTTCAC-3′ and IL-1β Forward 5′-ACAGATGAAGTGCTCCTTCCA-3′ and Reverse 5′-GTCGGAGATTCGTAGCTGGAT-3′ and glyceraldehyde-3-phosphate dehydrogenase (GAPDH) forward, 5ʹ-AGGGCTGCTTTTAACTCTGGT-3ʹ and GAPDH reverse, 5ʹ-CCCCACTTGATTTTGGAGGGA-3ʹ. PCR was performed under the following conditions: one cycle at 98°C for 3 min followed by 30–35 cycles at 95°C for 30 s, 55°C for 30 s, and 72°C for 30 s, with a final extension step at 72°C for 5 min. The amplified PCR products were analyzed via 2% agarose gel electrophoresis and EcoDye™ Nucleic Acid Staining Solution (Biofact Co., Ltd.); the relative intensities of the detected bands were measured on a Gel Doc2000 scanner (Bio-Rad, Hercules, CA, USA).

## Results

### LPS and PRP4 concomitantly induce cytokines expressions and rearrange cell actin cytoskeleton

**Figure 1.**
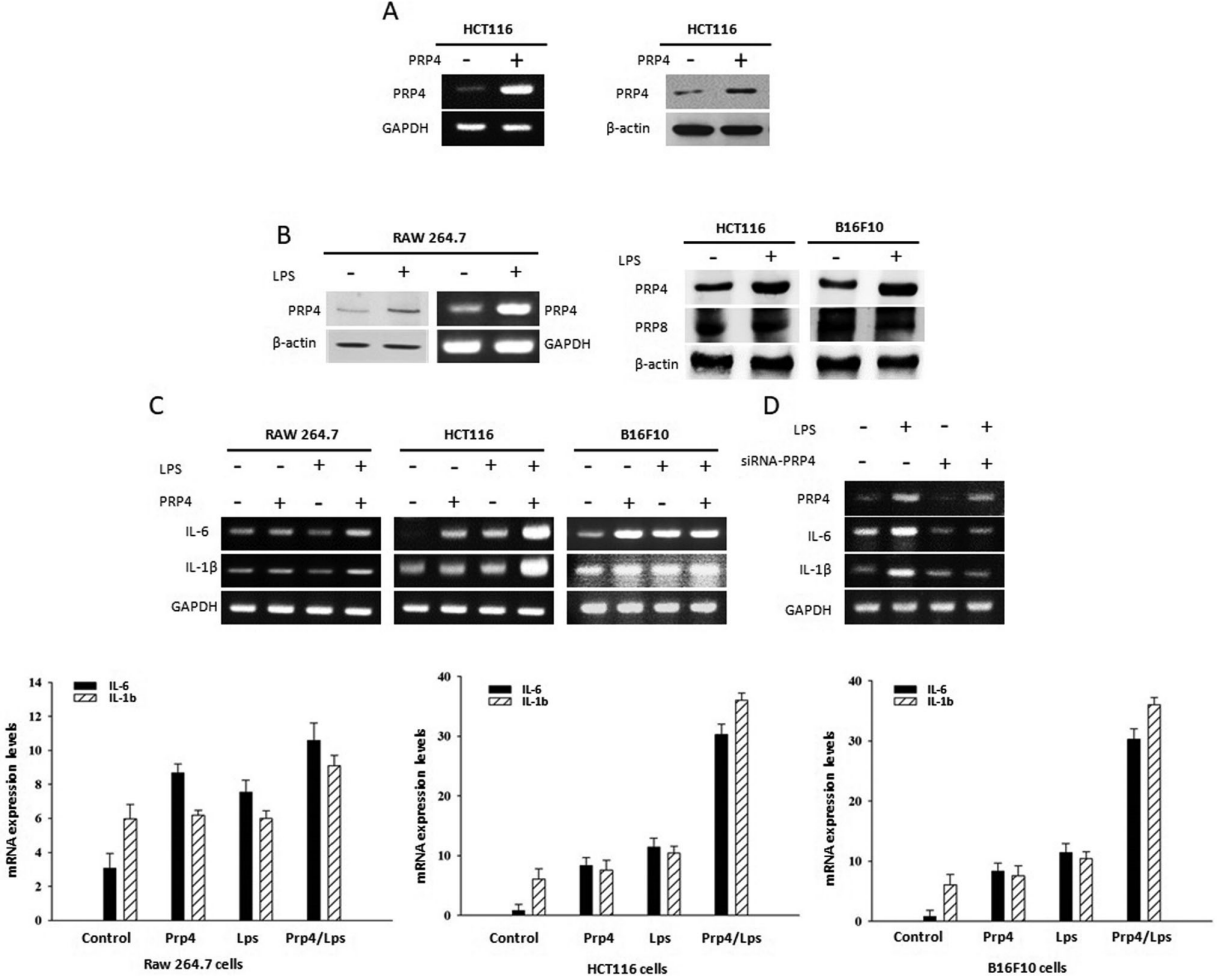
LPS and PRP4 induces cytokines expression. (A) mRNA and protein levels of PRP4 in control and PRP4-transfected cells. GAPDH and actin were used as the loading control. (B) Western blot and PCR analysis of PRP4 and PRP8 in Raw264.7, HCT116, B16-F10 cells after stimulation with 100 ng/ml LPS. (C) Cells were pre-treated with LPS, followed by transfection with PRP4, and then incubated for 24 h. RT-PCR was performed to examine the mRNA levels of IL-6 and IL-1β. GAPDH was used as internal control. (D) HCT116 cells were transfected with si-RNA-PRP4 using Xfect RNA transfection reagent from Takara as described by the manufacturer. Cells were then stimulated with 100 ng/ml LPS. RT-PCT and western blots were performed on control transfected cells. GAPDH was used as the loading control.

**Figure 2.**
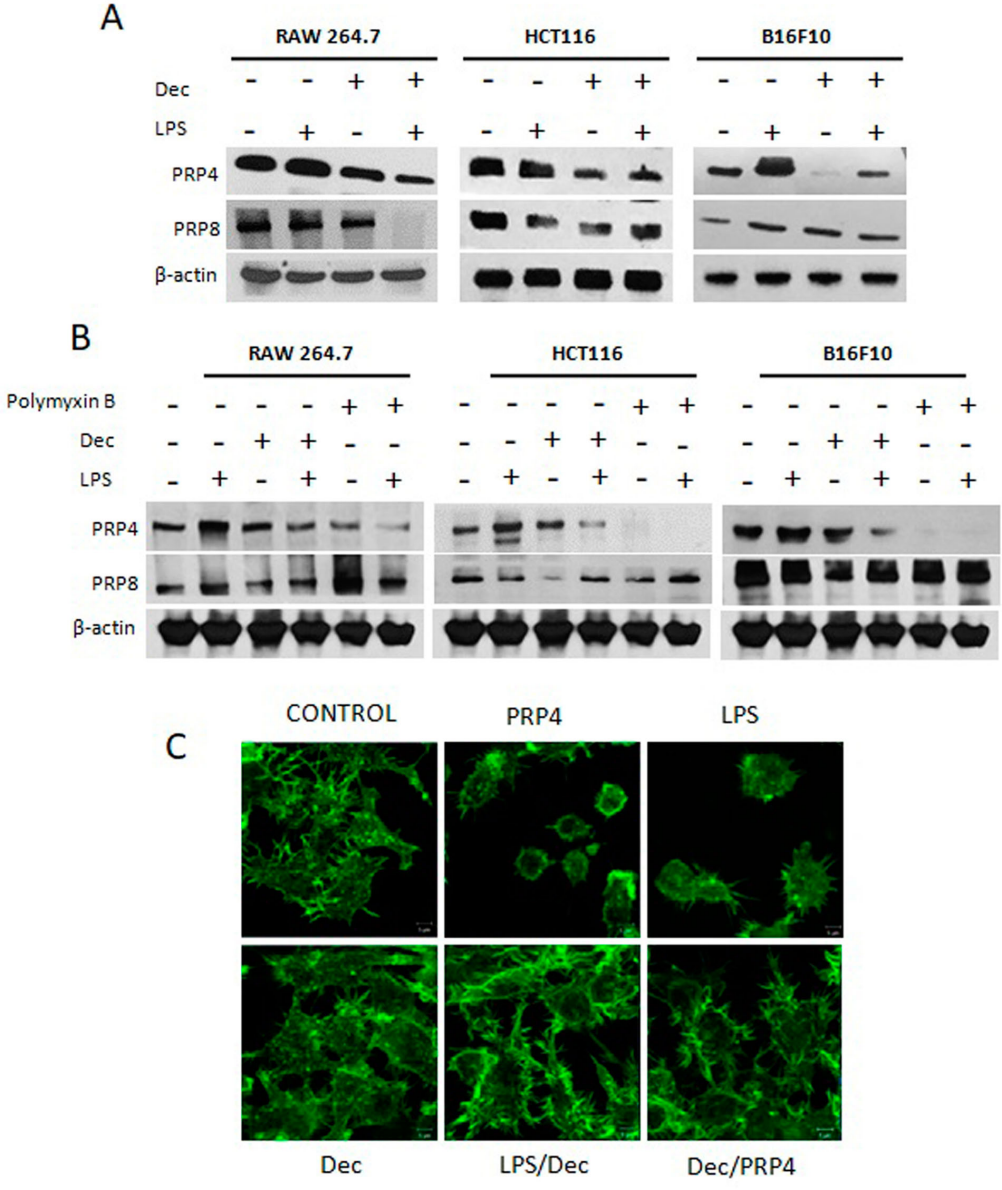
Decursin inhibits LPS-induced PRP4 expressions and cell morphological alterations. (A) Western blot analysis of PRP4 and PRP8 in Raw264.7, HCT116, B16-F10 cells after stimulation with 100 ng/ml LPS and treating with 10 µM decursin. β-actin was used as a loading control. (B) Cells were pre-treated with LPS and incubated with 10 µM decursin and/or 10 µg/ml polymyxin B for 24 h. Western blot was performed to analyze the expressions of PRP4 and PRP8. β-actin was used as a loading control. (C) Cells were stimulated with 100 ng/ml LPS, followed by transfection with PRP4, and treatment with 10 µM decursin. Cells were stained with phalloidin and observed under a ZEISS LSM 800 confocal microscope at 1000 × magnification.

HCT116 cells were transfected with a PRP4 expression construct, and PRP4 overexpression was confirmed both at the mRNA and protein levels by RT–PCR and western blotting, respectively (Figure 1(a)). It has been shown that when Gram-negative bacteria multiply in the host, LPS is released into the blood stream, where it triggers the induction of NF-κB-dependent proinflammatory cytokines such as prostaglandins, nitric oxide, tumor necrosis factor-α, and interleukin (IL)-1 (Triantafilou and Triantafilou 2005; Liu et al. 2017; Wassenaar and Zimmermann 2018). LPS has also been shown to induce inflammatory response in vitro (Lund et al. 2006). PRP4 has been shown to induce NF-κB signaling which probably prevented cancer cells from undergoing apoptosis (Islam et al. 2017). In order to investigate the relation between LPS and PRP4, we treated Raw 264.7 cells with 100 ng/mL LPS, and analyzed the expression of PRP4. We noted that LPS induced the expression of PRP4 both at protein and mRNA levels in Raw 264.7 cells. Similar results were obtained in HCT116 and B16-F10 cell lines. However, LPS did not induce the expression of PRP8 (Figure 1(b)). Next, we pre-treated the cells with LPS followed by PRP4 transfection, and observed that LPS and PRP4 concomitantly induced the expressions of pro-inflammatory cytokines IL-6 and IL-1β (Figure 1(c)). In order to confirm that LPS-induced cytokines activation is mediated through PRP4, we performed siRNA-mediated knockdown of PRP4 using a pool of three target-specific siRNAs, 19–25 nucleotides in length. Interestingly, siRNA-PRP4 also inhibits the LPS-induced overexpression of IL-6 and IL-1β (Figure 1(d)). Furthermore, by treating the cells with 10 µM decursin, it was revealed that LPS-induced PRP4 expression was blocked (Figure 2(a)). In order to confirm that LPS has induced PRP4 expression, we treated the cells with 10 µg/ml polymyxin B (LPS inhibitor) for 24 h, and observed the decreased expression of PRP4. However, PRP8 remained unaffected (Figure 2(b)). Previously, it has been shown that PRP4 altered cell morphology in cancer cell lines (Islam et al. 2017; Islam, Ahmed et al. 2018). In order analyze the correlation of LPS with PRP4 regarding cell cytoskeleton rearrangement, cells were pre-treated with LPS and transiently transfected with a PRP4 expression construct. Fluorescence confocal microscopy revealed that LPS and PRP4 over-expression induced actin filament redistribution and changed the cell morphology from an aggregated, flattened shape to a round shape, whereas cells incubation with 10 µM decursin reversed the induction of morphological changes (Figure 2(c)). These data suggest that decursin inhibits LPS-induced PRP4 expression, and thus blocks inflammatory response as well as cell morphology alterations.

### Decursin blocks LPS and PRP4-induced inflammatory pathway proteins

**Figure 3.**
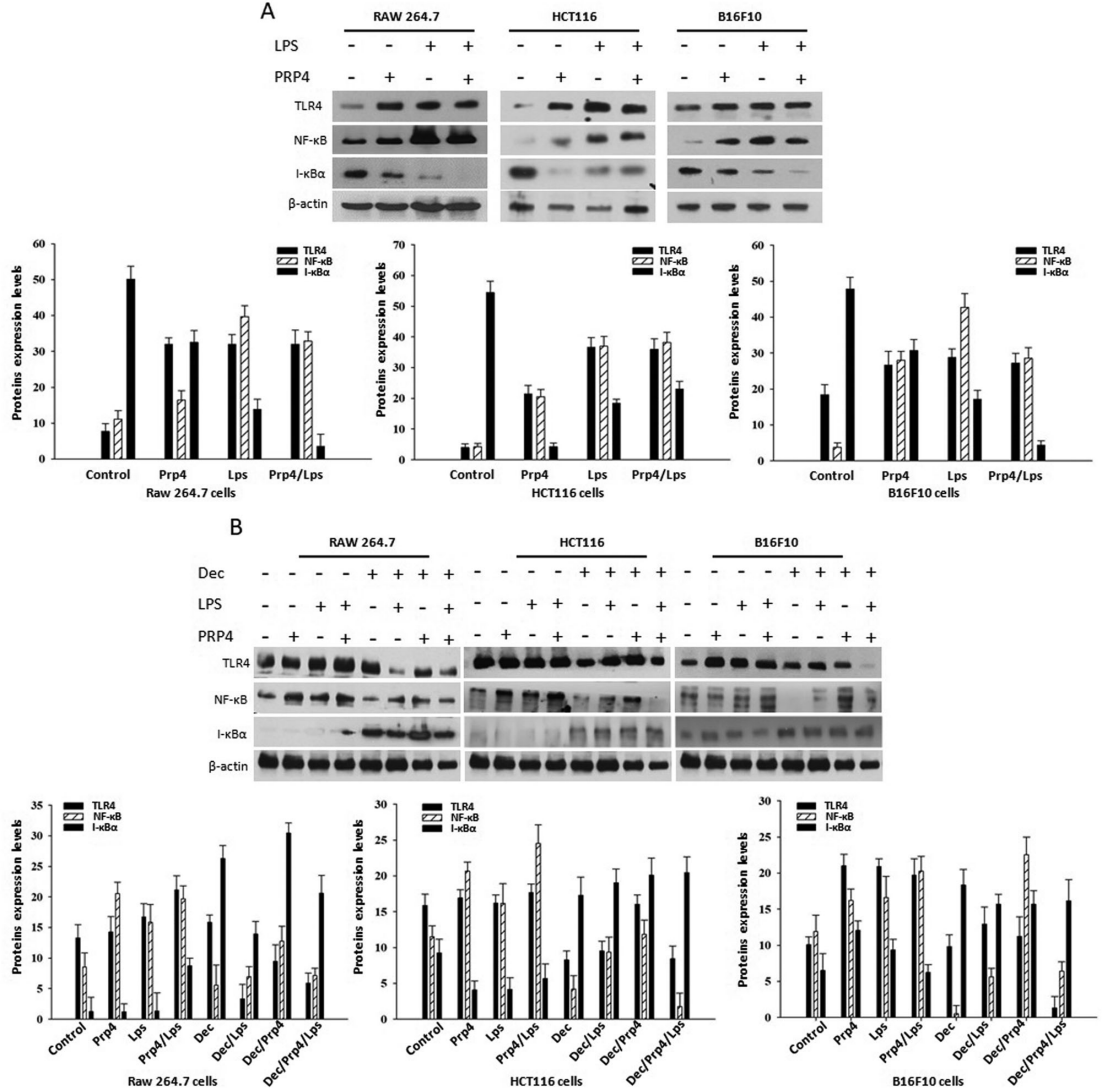
LPS and PRP4 induces inflammatory pathways proteins. (A) Cells were pre-treated with LPS and then transfected with PRP4. Cellular proteins were extracted using cell lysis buffer. Proteins were quantified by Bradford assay, and equal amount of proteins were separated on 10% SDS-PAGE. Proteins were then transferred onto nitrocellulose (NC) membranes. NC membranes were then incubated with specific antibodies for NF-κB, and I-κBα overnight at 4°C. Chemiluminescent signals were developed with Clarity™ ECL Western Blotting Substrate. (B) Western blot analysis of TLR4, NF-κB, and I-κBα after stimulating cells with LPS, followed by transfection with PRP4 and treatment with decursin. β-actin served as a loading control.

It has been well documented that LPS stimulates the activation of TLR4 in various cell lines (Guijarro-Muñoz et al. 2014). TLR4 is thought to share the MyD88-dependent pathway that activates NF-κB and mitogen-activated protein (MAP) kinases, and elevates the expression of genes encoding inflammatory cytokines (Kawai and Akira 2005; Lu et al. 2008). A recent study reported that TLR4-induced inflammation acted as a key promoter for cancer progression (Ran et al. 2019). It was reported that TLR4 was involved in facilitating migration of colon cancer cells and preserving them from immune surveillance and cell death (O’Leary et al. 2012; Tang and Zhu 2012). Additionally, it has been demonstrated that inhibition of TLR4 expression by rapamycin blocks TLR4/NF-κB signaling and promotes apoptosis of colon cancer (Sun et al. 2008). In order to investigate the concomitant effect of LPS and PRP4 on TLR4 and NF-κB signaling pathways, we pre-treated the cells with LPS followed by PRP4 transfection. Western blot analyses revealed that LPS and PRP4 upregulated the expression of TLR4 and NF-κB, while decreased the expression of inhibitory subunit I-κBα (Figure 3(a)). However, decursin treatment reversed the LPS and PRP4-induced activation of TLR4 and NF-κB (Figure 3(b)). These data suggested that LPS and PRP4 concomitantly induced the inflammatory response via activating TLR4/NF-κB signaling in the cells.

### LPS stimulated-PRP4 through activation of TLR4 and JNK is inhibited by decursin

**Figure 4.**
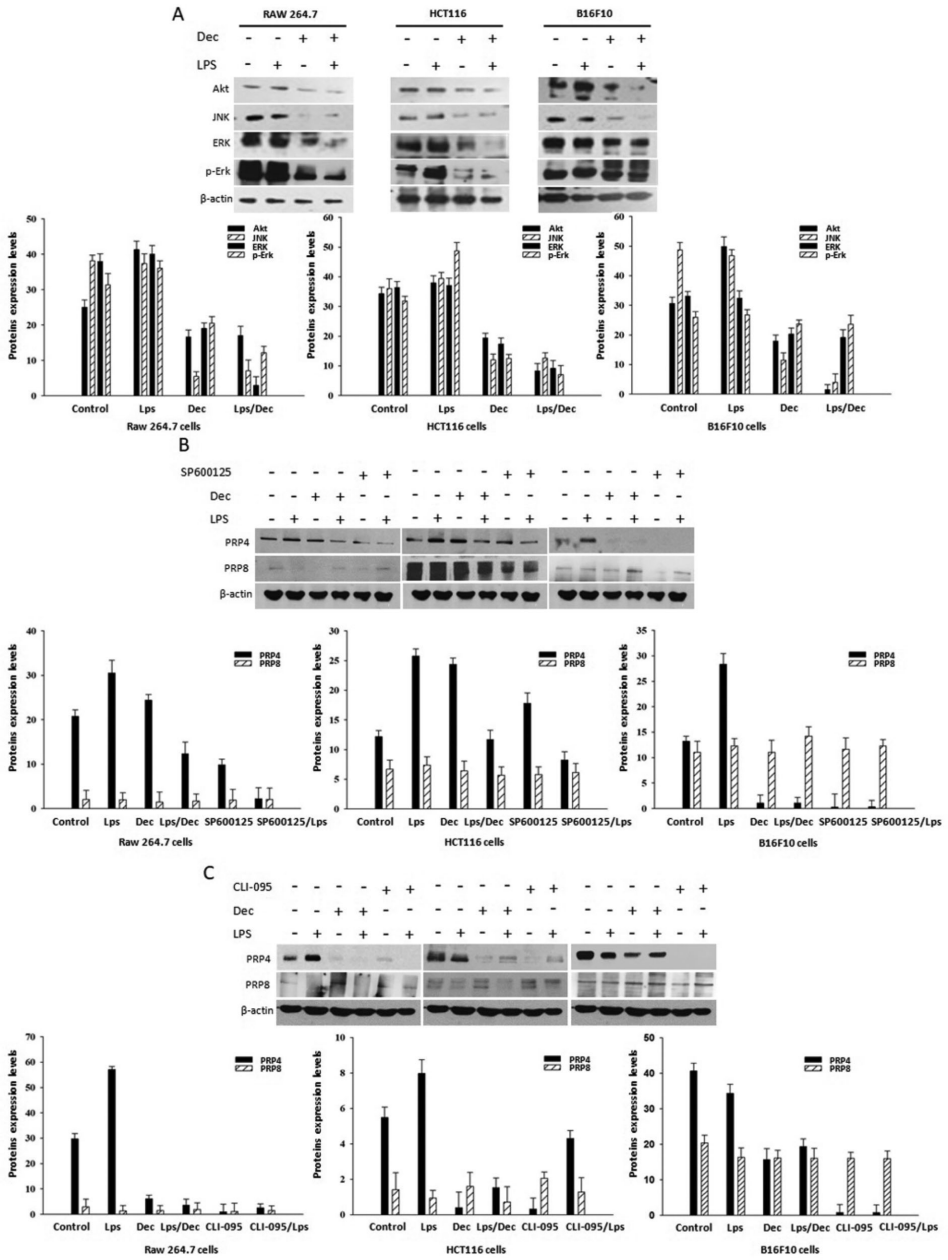
LPS stimulates PRP4 by activating TLR4 and JNK. (A) Protein levels of Akt, JNK, Erk, and p-Erk (B) PRP4 and PRP8 protein expressions were analyzed through western blot after incubating the cells with LPS, decursin and SP600125. (C) Cells were stimulated with 100 µg/ml LPS and incubated with or without 10 µM Decursin, and CLI-095 for 24 h. Protein levels of PRP4 and PRP8 were analyzed through western blotting. β-actin was used as a loading control.

Through western blot analyses, we observed that LPS induced the expressions of Akt, JNK, ERK, and p-ERK in Raw 264.7, HCT116, B16-F10 cells, which were then blocked by concomitant treatment of 10 µM decursin (Figure 4(a)). In order to investigate the involvement of TLR4 and JNK pathway in LPS induced-PRP4 expression, we utilized the inhibitors for TLR4 (CLI-095) and JNK (SP600125) along with LPS and decursin. CLI-095 is a cyclohexene derivative that specifically suppresses TLR4 signaling, inhibiting the production of nitric oxide and pro-inflammatory cytokines (Ii et al. 2006). CLI-095 acts by blocking the signaling mediated by the intracellular domain of TLR4, and has been shown to potently suppress both ligand-dependent and -independent signaling of TLR4 (Kawamoto et al. 2008). SP600125, an anthrapyrazolone, is a novel and selective inhibitor of JNK that competes with ATP to inhibit the phosphorylation of c-Jun. It prevents the activation of inflammatory genes such as COX-2, IL-2 IFN-γ and TNF-α (Bennett et al. 2001). Interestingly, we found that CLI-095 and SP600125 treatment remarkably blocked LPS-induced PRP4 expression, while it did not affect PRP8 expression (Figure 4(b and c)). These results suggest that PRP4 activation by LPS is associated with upregulation of TLR-4 and JNK.

## Discussion

In this study, we have shown that LPS induced the expression of PRP4 in Raw 264.7, HCT116, and B16-F10 cell lines. Both the LPS and PRP4 induced the expression of pro-inflammatory cytokines like IL-6 and IL-1β. Additionally, LPS and PRP4 increased the expressions of inflammatory pathways proteins like TLR4 and NF-κB. Decursin blocked the LPS-induced PRP4 expression as well downregualted the TLR4 and NF-κB. Finally, we established a possible link of LPS with TLR4 and JNK, through which it activated PRP4.

LPS has been shown to stimulate host cells such as macrophages to produce endogenous mediators including cytokines, prostaglandins, and nitric oxide. MAPKs including JNKs, ERKs, p38 MAPKs and ERK5 have been reported to be stimulated by LPS (Zhu et al. 2000). JNK has been shown to play a special role in mediating LPS responses in macrophages by phosphorylating transcription factors including c-Jun and ATF-2, which then activate the transcription of iNOS, COX-2, and various other inflammatory cytokines (Rao 2001).

Studies have shown that most of the LPS signals are mediated by TLR4 (Lu et al. 2008; Hsu et al. 2011). TLRs recognize molecular patterns of invading pathogens. A study reported that TLR4 was essential for LPS-mediated JNK activation, as JNK activation was abrogated in mice with TLR4 null-functional mutation (Muzio et al. 1998). It was shown that MyD88, which directly associates with the cytoplasmic domain of TLRs, was needed for the early activation of JNK by LPS, because JNK activation was delayed in macrophages of MyD88-deficient mice (Kawai et al. 1999).

During our investigation, to confirm that PRP4 was stimulated by LPS, we utilized polymyxin B for inhibiting LPS action. Polymyxin B is a potent decapeptide cyclic cationic antibiotic, containing lipophilic and hydrophilic groupment (lipophobic), that binds to the lipid A portion of LPS and neutralizes it (Tsuzuki et al. 2001; Ferrari et al. 2004). Cells incubated with polymyxin B and decursin showed decreased expressions of PRP4.

In order to find whether LPS was having some link with the PRP4 stimulation through the activation of TLR4 and JNK, we utilized the inhibitors for TLR4 (CLI-095), and JNK (SP600125). Interestingly, PRP4 activation was blocked with these inhibitors. We can assume from these findings that TLR4 and JNK served as upstreamregulators for PRP4. At the same time, we noted that PRP8 was not affected either by polymyxin B or by CLI-095 and SP600125. We have no clear clue about this phenomenon; however it may be due to the fact that PRP4 contains a kinase domain (amino acid sequence 687–1003) which shares homology with cyclin-dependent kinases and MAPKs (Kojima et al. 2001; Lützelberger and Käufer 2012). It is possible that PRP4 got activation by TLR4 and JNK pathway proteins by interacting through its kinase domain. However, further investigations are needed for the confirmation of this mechanism. As PRP8 lacks the kinase domain, it may be the reason that it remained unaffected by polymyxin B, CLI-095, and SP600125.

Our study provides molecular insights for LPS-related pathogenesis and a basis for developing new strategies against metastasis in colorectal cancer and skin melanoma. Additionally, it is emphasized that decursin may be an effective treatment strategy for various cancers, including colorectal and skin melanoma, in which LPS and PRP4 lead to the induction of inflammatory response.
